# The impact of perceived work dirtiness on nursing students’ professional commitment: the mediating role of career adaptability and the moderating role of social support - a cross sectional study

**DOI:** 10.1186/s12912-024-01963-4

**Published:** 2024-05-02

**Authors:** Chen Jilong, Tao Yueying, Chen Huizheng, Meng Yong, Li Genqiang

**Affiliations:** 1 School of Health Management, Xinxiang Medical University, Xinxiang, 453003 China; 2 The Second Affiliated Hospital of Xinxiang Medical University, Xinxiang, 453002 China

**Keywords:** Nursing student, Perceived work dirtiness, Career adaptability, Professional commitment, Social support

## Abstract

**Background:**

Social consensus in the nursing industry is that the job is accompanied by dirtiness. It is generally accepted that negative cognition about a career is an important determinant in reducing nursing students’ professional commitment. However, the impact of nursing students’ perceived work dirtiness on professional commitment and its mechanism remains unclear. This study aimed to analyze the association between perceived work dirtiness and professional commitment and to examine the mediating role of career adaptability and the moderating role of social support among nursing students in mainland China.

**Methods:**

A cross-sectional online study was conducted among 341 nursing students from three medical universities in Henan, China. The participants’ perceived work dirtiness, career adaptability, professional commitment, and social support were obtained. SPSS 26.0 and Amos 23.0 software were used for the statistical analysis.

**Results:**

Perceived work dirtiness was negatively related to career adaptability and professional commitment (*P* < 0.001). Career adaptability played a partial mediating role (*β*= -0.177, *P* < 0.001) in the relationship between perceived work dirtiness and professional commitment. Social support played a moderating role (*β* = 0.134, *P* < 0.01) in the relationship between perceived work dirtiness and career adaptability. Moreover, social support moderates the mediating role of career adaptability.

**Conclusions:**

Nursing students’ perceived work dirtiness is an important factor in reducing professional commitment. Therefore, nursing educators must enhance courses focusing on professional qualities, providing avenues for nursing students to access social support. This proactive approach aims to mitigate the adverse impact of perceived work dirtiness on professional commitment among nursing students.

As China has an aging population and escalating healthcare demands, a deficit of 2 million nursing personnel looms large [1]. Driven by this significant demand, nursing students, as vital reserves for future healthcare professionals, pose a critical concern for nursing educators: how to cultivate their professional commitment [2]. Professional commitment is individuals’ attitude towards and identification with their profession or career, it reflects the level of drive individuals exhibit in pursuing goals within their professional domain [3]. Researches indicate that if nursing students possess higher levels of professional commitment, they can maintain this commitment once they become registered nurses in hospitals and are willing to engage in the nursing profession [4]. In the long run, this ensures the effectiveness and sustainability of nursing professionals. Furthermore, professional commitment can be cultivated during university education and is closely intertwined with nursing students’ understanding of their profession, capabilities, and interpersonal relationships [5, 6]. Scholars emphasize that shaping nursing students’ professional commitment should permeate the entire university education process [4]. Given the stability and malleability of professional commitment, identifying factors influencing nursing students’ professional commitment has significant practical implications for nursing education.

The current research landscape heavily focuses on examining the positive factors influencing nursing students’ professional commitment [2, 7, 8], leaving a noticeable gap in understanding the impact of negative psychological traits on their professional commitment. In recent years, perceived work dirtiness has received researchers’ attention. The heightened perceived work dirtiness (PWD) is usually associated with various detrimental career adaptation results, such as a diminished sense of social value and professional identity [9, 10]. However, the relationship between perceived work dirtiness and nursing students’ professional commitment remains unexplored. From a practical perspective, Nursing students can realize that the nursing profession is surrounded by dirtiness, and called it dirty work [11, 12]. Meanwhile, Career construction theory suggests that individual’s perceptions of career/profession play a crucial role in determining career adaptation outcomes (professional commitment) [13, 14]. This highlights the possibility and necessity to further explore the relationship between perceived work dirtiness and nursing students’ professional commitment through the lens of career construction theory [11]. . To address this gap, the primary aim of the current study is to explored the relationship between perceived work dirtiness and professional commitment.

How does perceived work dirtiness affect nursing students’ professional commitment? Career adaptability, a core element of the career construction theory, is individual’s readiness to adjust to various work responsibilities and role transitions [14]. Scholars have found that negative perceptions associated with one’s career/profession can have adverse effects on career adaptability [15, 16]. The more difficulties students perceive in their current or future professional endeavors, the lower their career adaptability. Simultaneously, empirical research consistently demonstrates a stable positive association between career adaptability and professional commitment [17]. Therefore, we propose that career adaptability may mediate the relationship between perceived work dirtiness and professional commitment.

Social support may be the boundary condition between perceived work dirtiness and career adaptability. Nursing students are currently in the learning phase, and their life environment is composed of family, teachers, and peers. The literature indicates that college students who receive support from peers and teachers are better equipped to navigate challenging situations [18]. Social support refers to the assistance individuals receive from their surrounding environment, including school and family [19]. The career adaptation model suggests that the environment could be an important factor affecting the career construction process and that career adaptability results from the integration of individual-environment interactions [14]. Hence, aligned with career construction theory, this study suggests that social support from family, teachers, and significant others may moderate the relationship between perceived work dirtiness and nursing students’ career adaptability. Building upon this, we also investigated the mediating role of career adaptability and the moderating role of social support to unveil the underlying mechanisms of the relationship between perceived work dirtiness and professional commitment.

## Relationship between perceived work dirtiness and professional commitment

Perceived work dirtiness is individuals’ subjective perceptions of undignified work and its environment [20]. This primarily involves bodily aspects (related to death, waste disposal, and life-threatening environments), social dimensions (associated with individuals or groups involved in crime, disease, and feelings of degradation), and moral considerations (linked to deviations from ethical norms, privacy infringements, and behaviors contravening civilized standards) [21]. Using the nursing profession as an illustrative example, inherent nursing responsibilities include contact with patients and the handling of patient excretions, potentially inducing a sense of bodily dirtiness among nursing students not yet employed [22]. In addition, in terms of social relations within the healthcare industry, nurses often experience a lower social status. In the context of nursing duties, nurses may find themselves subjected to verbal abuse and challenges from patients’ families, thereby potentially inducing a sense of social dirtiness among nursing students [23]. Furthermore, nurses may grapple with moral dilemmas related to life and death. For example, the medical team can provide treatment to the patient, but the patient’s family may eventually decide to stop the treatment, thereby eliciting a sense of moral dirtiness [24]. Lv have found that perceived work dirtiness may hinder the future career development of hotel management students, even lead them to abandon their original major [25].

Professional commitment is individuals’ attitude towards and identification with their profession or career [3]. The research suggests that professional commitment contributes to increased student engagement, leading to better academic performance [26]. Because it manifests the students’ sense of identification with their current major and their sustained positive attitude toward engaging in their major. Professional commitment is typically influenced by students’ cognitive perceptions of the career attributes [5]. Research by Chen (2023) has shown that occupational stigma could lead individuals to adopt protective measures [27]. Nursing students may mitigate the impact of work-related stress on their resources by diminishing their sense of professional commitment [28]. Perceived work dirtiness is also a negative perception of nursing students toward the profession. Career construction theory emphasizes individuals’ perceptions of career/profession could exert an important influence on career adaption outcomes [14]. Drawing from career construction theory, The quasi-subordinate relationship between nurses and patients, which involves social dirtiness, could causes nursing students to face pressure from the loss of individual psychological resources [29]. And this pressure prompting them to adopt various avoidance strategies, such as diminishing professional commitment or changing their career path, to safeguard personal resources [30]. Prior research has revealed that factors related to perceived work dirtiness, including contact with pollutants and perceived discrimination, notably predict a reduction in professional commitment [31, 32]. In addition, due to lower social status, the demanding of the profession, the high workload, and the existence of night shifts in nursing, students may attribute negative work experiences to career attributes, thereby strengthening the negative impact of perceived work dirtiness on professional commitment [5, 33]. Therefore, we propose the following hypothesis:

### H1

Perceived work dirtiness negatively predicts professional commitment in nursing students.

## The mediating role of career adaptability

Career adaptability, which is individual’s readiness for self-adjustment in diverse work responsibilities and role transitions, includes four dimensions: career concern, career control, career curiosity, and career confidence [34]. These dimensions collectively address pivotal career development inquiries: “Do I have a future?” “Who owns my future?” “What do I aspire to achieve in the future?” and “Am I capable of realizing these aspirations?” [35]. These facets constitute fundamental competencies enabling individuals to effectively solve a series of career challenges inherent in their career development and role changes and grapple with broader career-related issues [14, 35]. Researches focusing on samples of college students have shown that individuals with high levels of career adaptability not only possess a strong vocational self-efficacy but also have the capacity to promptly adjust their career mindset and attain satisfactory career outcomes [36].

According to career construction theory, perceived work dirtiness potentially decreases nursing students’ career adaptability. Researches posit that the experience of perceived work dirtiness may subject students to stress and emotional perturbations associated with their future professional trajectory [12]. Given that nursing students are in the early stages of career development, these stresses and perturbations might impede their loss of curiosity and concern about career construction [37, 38]. The consequence is a potential limitation in the cultivation of their career construction abilities, which constrains a comprehensive grasp of the challenges and opportunities to the nursing profession [39]. Moreover, rigorous academic demands, information overload, and the exact nature of clinical training often cause academic stress for nursing students [40]. Confronted with these external, volatile, and uncontrollable pressures, perceived work dirtiness may have a negative impact on career confidence and career control. This leads to a significant decline in career adaptability [41].

The extant research has successfully established a positive correlation between career adaptability and professional commitment [7, 17, 42]. According to career construction theory, career adaptability is an adaptive resource used to shape adaptive responses [35]. Professional commitment has already been identified as a highly adaptive response that is positively influenced by the increase in career adaptability [42]. Specifically, high career adaptability means that nursing students pay more attention to their career construction, which may prompt them to explore the professional value of the nursing industry and combine it with the realization of personal value [43], thereby nurturing a more profound emotional affinity and motivational impetus toward their professional—commonly known as professional commitment. Longitudinal investigations substantiate this, indicating that nursing students with high career adaptability tend to exhibit sustained increases in professional commitment over time [4]. Therefore, we propose the following hypothesis:

### H2

Career adaptability plays a mediating role in the effect of perceived work dirtiness and professional commitment.

## The moderating role of social support

Social support is the assistance individuals receive from their surrounding environment, including school and family [19]. Career construction theory suggests that the development of career adaptability is influenced by environmental factors, positive situational factors can assist individuals in effectively addressing the impacts of vocational challenges, facilitating positive adaptation outcomes [14]. For instance, Ataç’s research found that social support among young adults moderates the relationship between self-esteem and career adaptability [44]. Therefore, in alignment with career construction theory, the present study posits that social support is likely to impact the strength of the relationship between nursing students’ perceived work dirtiness and career adaptability. Nursing students with high levels of social support typically exhibit greater confidence in their future career development. They acknowledge the presence of perceived work dirtiness while maintaining a positive perspective on its relationship with social identity and career advancement [45]. As a result, they persistently engage in career preparation efforts [16, 46], thus mitigating the negative effects of perceived work dirtiness on career adaptability [38]. Conversely, nursing students with limited social support experience increased pressure and insecurity when faced with perceived work dirtiness, amplifying its negative impact on career adaptability.

Furthermore, nursing students with high social support tend to exhibit relatively higher career adaptability when facing perceived work dirtiness. This could further mitigate the negative impact of perceived work dirtiness on professional commitment. Conversely, nursing students with low social support experience lower career adaptability when confronted with perceived work dirtiness, leading to lower levels of professional commitment. Therefore, we hypothesize that social support can mitigate the negative effects of perceived work dirtiness on career adaptability and the indirect effect of perceived work dirtiness on professional commitment.

### H3

Social support plays a moderating role in the relationship between perceived work dirtiness and career adaptability.

### H4

The indirect effect of perceived work dirtiness on professional commitment via career adaptability is moderated by social support such that the indirect effect is stronger when social support is low, but weakens when social support is high.

## Method

### Study design

A correlational cross-sectional study was performed to explore how career adaptability acts as a mediator in the link between nursing interns’ perceived work dirtiness and their professional commitment, and how social support acts as a moderator in the link between nursing students’ perceived work dirtiness and their career adaptability.

### Participants

To achieve a representative sample, cluster sampling was employed in this research. Initially, three medical universities in different cities in China were selected. Nursing undergraduates meeting the inclusion criteria were recruited, which included (a) full-time students, (b) enrolled in the Bachelor of Science in Nursing Program, (c) aged between 18 and 24 years, (d) fluent in Chinese, and (d) those who provided informed consent for voluntary participation. Nursing students who participated in nursing internship courses for less than eight months were excluded.

### Data collection

For data collection, an online survey platform (Wenjuanxing) was utilized. Teaching managers, who serve as the primary investigators, agreed to collaborate and undergo relevant training. Before the official survey, a presurvey with a 5% sample size was conducted to ensure questionnaire clarity. After making minor adjustments, the formal survey was initiated, two items are employed to assess whether participants are responding attentively (“My birthday is on February thirty-first” and “I will choose 3 for this item”), and information was disseminated through online student work groups. Students were initially provided with written explanations regarding the study’s objectives, procedures, principles of voluntary participation, and anonymity. They were given the choice to opt out or withdraw without any impact on their academic performance. Upon agreeing to participate and return of signed electronic informed consent forms, the participants were instructed to truthfully complete the questionnaire, which typically took approximately 10 min. All collected data were encoded after excluding questionnaires that did not pass the test items, had excessively short response times, or selected the same number in more than two scales.

### Data analysis

The statistical analyses were conducted using IBM SPSS version 26.0. Enumeration data are presented as frequencies and composition ratios, while measurement data are presented as the means and standard deviations. Variations in professional commitment among students with different characteristics were compared through two independent sample t-tests and one-way ANOVA. Pearson’s correlations were employed to discern associations between variables. To examine the mediating effect of Career Adaptability, Amos 23.0 was utilized, and the bootstrap method was applied to ascertain the significance of the mediating and moderating effects. The 5000 bootstrapped samples method with a 95% bias-corrected confidence interval (CI) estimation was employed to evaluate whether career adaptability mediated the link between perceived work dirtiness and professional commitment [47]. A significance level of *P* ≤ 0.05 was considered to indicate statistical significance.

### Calculation of the sample size

Participants were conveniently sampled from nursing students at three colleges in H province, China. Using the G*Power 3.1.9.2 program, the number of research participants was calculated with a significance level (α) of 0.05, a power (1-β) of 0.95, an effect size (f2) of 0.15, and six predictors. The resulting sample size was 146. The data were collected from 400 participants considering a dropout rate of 15%.

### Measure

A structured survey instrument encompassing five distinct sections was used for data collection: demographic questionnaire, Scale for Perceived Work Dirtiness (PWDS), Career Adapt-Abilities Scale (CAAS), Scale for Professional Commitment (PCS), and Social Support Scale (SSS).

### Perceived work dirtiness

Perceived work dirtiness was assessed using Lai et al.‘s 12-item scale [48]. Participants expressed their sentiments regarding job characteristics on a scale from 1 (strongly disagree) to 5 (strongly agree). An example item was “I had to handle certain things that were dirty in the course of my duties.” The Cronbach’s α coefficient was 0.863.

### Career adaptability

The career adaptability scale was developed by Savickas et al. [13] and was translated by Hou [49]. The 24-item scale is categorized into four dimensions: concern, control, curiosity, and confidence. Each item is rated on a 5-point Likert scale (ranging from 1 = strongly disagree to 5 = strongly agree). In this study, the Cronbach’s α coefficient for the Chinese version was 0.967. Additionally, the Cronbach’s α coefficients for each dimension were 0.884, 0.894, 0.900, and 0.906.

### Professional commitment

The professional commitment scale was developed by Blau [3]. The professional commitment scale is an 8-item measure of one’s attitude toward one’s profession/vocation – that is, an individual’s level of commitment to a profession or career field (e.g., ‘I like this profession too much to give it up’). The items are rated from 1 (strongly disagree)–5 (strongly agree). The reliability of professional commitment in this study reached a Cronbach’s alpha of 0.868.

### Social Support

We assessed social support with Xiao’s 12-item scale [50]. The scale comprises 12 items, with response choices ranging from 1 point (strongly disagree) to 7 points (strongly agree). The SSS evaluates the quality of social support in three areas: family, teachers, and significant others. The Chinese version of the SSS has demonstrated robust reliability and validity across various Chinese populations, with a Cronbach’s alpha of 0.936. Furthermore, the Cronbach’s α coefficients for each dimension were 0.867, 0.872, and 0.871, respectively.

### Check for common method bias

Given that all data in this study originated from self-reports by nursing students, the possibility of common method bias exists. To mitigate and control this potential bias and enhance the rigor and validity of the research results, precautionary measures were implemented. These measures included ensuring anonymous responses, informing participants that all information is strictly confidential and used solely for scientific research, reverse scoring of individual items, and clarifying ambiguous statements [51]. Additionally, Harman’s single-factor test was applied to assess common method bias. The results indicated that the eigenvalues of all four factors exceeded 1, and the variance explained by the first factor was 25.93%, which was well below the critical threshold of 40% [52]. This outcome suggests the absence of significant methodology bias in the study.

## Results

### Participants’ demographic characteristics and their distribution by professional commitment score

The sample comprised a total of 342 individuals, with 28.4% being males and 71.6% being females. The average age was 22.13 ± 0.87 years, ranging from 19 to 24 years. Regarding study hours per day, 27.8% were dedicated to 0 to 2 h, 32.5% spent 2 to 4 h, 23.1% allocated 4 to 6 h, and 16.7% invested more than 6 h. Only 37.7% had relatives or friends in the nursing industry, while 62.3% did not. No significant differences in professional commitment scores emerged based on gender and the time for studying each day. Nevertheless, significant variations were observed in some variables between groups defined by age and relatives or friends who were nurses (Table 1).

**Table 1:** Participants’ demographic characteristics and their distribution by professional commitment score (*N* = 342)

Characteristics	*N*(%)	Professional commitment		
		Mean ± SD	t/F	*P*
**Gender**				
Male	97(28.4)	30.41 ± 5.01	1.58	0.209
Female	245(71.6)	29.51 ± 6.31		
**age**				
≤ 21	77(22.5)	33.22 ± 4.09	16.61	< 0.001
22	168(49.1)	32.69 ± 6.27		
≥ 23	97(28.4)	31.52 ± 5.56		
**Time for studying each day**				
0 ~ 2 h	95(27.8)	28.84 ± 6.33	1.55	0.202
2 ~ 4 h	111(32.5)	30.20 ± 5.63		
4 ~ 6 h	79(23.1)	30.58 ± 5.53		
More than 6 h	57(16.7)	29.33 ± 6.52		
**Relatives or friends is nurses**				
Yes	129(37.7)	31.26 ± 5.16	39.11	< 0.001
No	213(62.3)	27.30 ± 6.42		

### Mean scores and correlations among PDW, CAA, PC, and social support

The average scores of the nursing students were 3.20 ± 1.21 for perceived work dirtiness, 3.99 ± 0.65 for career adaptability, 3.72 ± 0.75 for professional commitment, and 5.27 ± 0.98 for social support. The correlations between perceived work dirtiness, career adaptability, professional commitment, and social support revealed that perceived work dirtiness was negatively associated with career adaptability and professional commitment (*r*=-0.450, *p* < 0.001; *r*=-0.395, *p* < 0.001, respectively). Career adaptability was positively associated with professional commitment (*r* = 0.661, *p* < 0.001) (Table 2).

**Table 2:** Mean scores and correlations among the study variables (*N* = 342)

Variable	Mean ± SD	1	2	3	4
**1.Perceived work Dirtiness**	3.20 ± 1.21	1			
**2.Career adaptability**	3.99 ± 0.65	-0.450^**^	1		
**3.Professional commitment**	3.72 ± 0.75	-0.395^**^	0.661^**^	1	
**4.Social support**	5.27 ± 0.98	-0.460^**^	0.688^**^	0.463^**^	1

* *P* < 0.05; ** *P* < 0.01

### Hypothesis test

Initially, a series of confirmatory factor analyses were conducted, comparing the four-factor model (with PDW, CAA, SS, and PC as distinct factors) with various alternatives. The confirmatory factor analysis of this model demonstrated a favorable fit for the data (χ²/df = 2.56, CFI = 0.96, TLI = 0.97, RMSEA = 0.06, and SRMR = 0.03).

Subsequently, we examined the mediating effect of career adaptability on the relationship between perceived work dirtiness and professional commitment. Amos 23.0 was utilized construct a structural equation model and conduct percentile bootstrap bias-corrected resampling with 5000 samples for mediation effect testing. The results indicated that all model fit indices met the evaluation criteria (χ²/df = 3.51, CFI = 0.95, TLI = 0.95, RMSEA = 0.07, and SRMR = 0.05), and the path coefficients of the mediation model were estimated using the maximum likelihood method (ML). The results indicated that perceived work dirtiness negatively predicted professional commitment (*β* = -0.174, *t* = -4.15, *p* < 0.001). Moreover, perceived work dirtiness had a significant negative predictive effect on career adaptability (*β* = -0.428, *t* = -8.76, *p* < 0.001), and career adaptability had a significant positive predictive effect on professional commitment (*β* = 0.576, *t* = 13.68, *p* < 0.001) (Table 3). The upper and lower bounds of the bootstrap 95% confidence intervals (bootstrap 95% CI) of the direct impact of perceived work dirtiness on professional commitment and the mediating effect of career adaptability did not contain 0 (Table 3), which showed that perceived work dirtiness could predict professional commitment not only directly but also indirectly through the mediating effect of career adaptability (Boot SE in Table 3 refers to the standard error of the indirect effects estimated by the bias-corrected percentile bootstrap method). The direct effect (-0.174) and mediation effect (-0.247) accounted for 41.33% and 59.67% of the total effect (-0.421), respectively. Hypothesis 2 was supported.

**Table 3:** Mediation model and effect (*N* = 342)

Path		Informant				
		β	SE	*p*	95% CI	
c (total effect)	Perceived work dirtiness → Professional commitment	-0.426	0.047	< 0.001	-0.519	-0.334
a	Perceived work dirtiness → Career adaptability	-0.434	0.049	< 0.001	-0.530	-0.338
b	Career adaptability → professional commitment	0.574	0.042	< 0.001	0.490	0.656
c’ (direct effect)	Perceived work dirtiness → Professional commitment	-0.177	0.042	< 0.001	-0.260	-0.094
Indirect effect	Perceived work dirtiness → Career adaptability → Professional commitment	-0.249	0.040	< 0.001	-0.349	-0.162

**Fig. 1:**
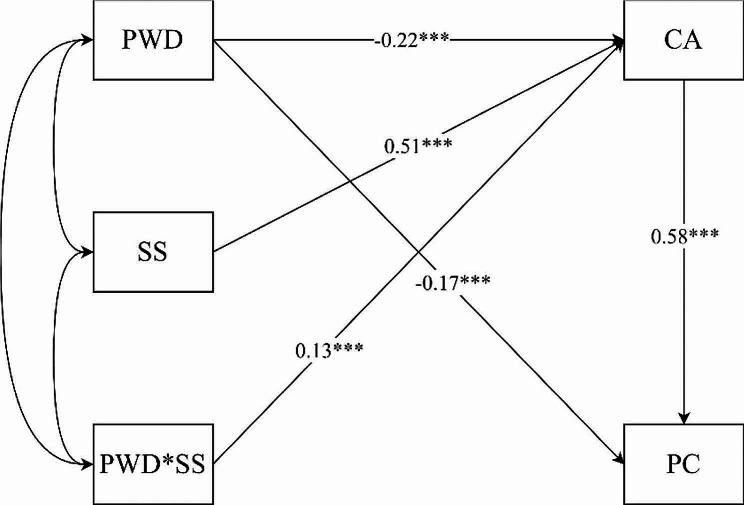
Moderated mediation model. ***p < 0.0001, **p < 0.001, *p < 0.05 PWD: Perceived work dirtiness; CA: career adaptability; PC: Professional commitment; SS: Social support

Next, to test whether the influence of perceived work dirtiness on professional commitment through career adaptability was moderated by social support. And to comprehensively examine the relationships between variables and mitigate the interference of measurement errors on research findings, structural equation modeling was used to test the relationships among variables. The means of all variables was centered and the interaction terms between perceived work dirtiness and social support was generated. Subsequently, structural equation models were constructed with perceived work dirtiness as the independent variable, professional commitment as the dependent variable, career adaptability as the mediating variable, and social support as the moderating variable. The results indicated that all model fit indices met the evaluation criteria (χ²/df = 3.68, CFI = 0.95, TLI = 0.93, RMSEA = 0.07, and SRMR = 0.06) The path relationships between the variables are shown in Fig. 1. After social support was entered into the model, the results showed that the interaction of perceived work dirtiness and social support had a significant predictive effect on career adaptability (*β* = 0.135, *t* = 4.26, *p* < 0.01), which indicates that social support moderates the first half of the mediating process. Hypothesis 3 was confirmed.

**Fig. 2:**
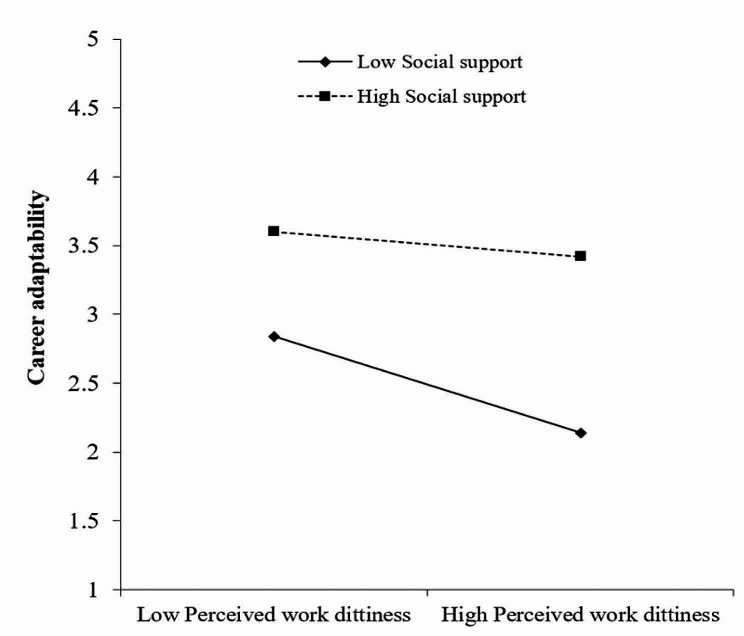
Moderating effect of social support on the relationship between perceived work dirtiness and career adaptability

Finally, to elucidate how social support adjusts for the effect of perceived work dirtiness on career adaptability, we conducted a simple slope test (Fig. 2). The results showed that when the level of social support was low (M-1SD), the impact of perceived work dirtiness on career adaptability was significant (*β* = -0.352, *t* = -6.45, *p* < 0.001). When the level of social support was relatively high (M + 1SD), the negative effect of perceived work dirtiness on career adaptability was significant (*β* = -0.110, *t* = 2.35, *p* < 0.001). In addition, at three levels of social support, career adaptability also showed a decreasing effect on the relationship between perceived work dirtiness and professional commitment; that is, as the social support of nursing students increased, the negative predictive effect of perceived work dirtiness on career adaptability gradually weakened.

This study further used the Monte Carlo simulation method to test the moderated mediation effect model of this study. The results showed (Table 4) that when the level of social support was low (M-1SD), the indirect effect was significant (*β* = -0.203, BootSE = 0.046), and the bootstrap 95% CI of the indirect effect did not contain 0. When the level of social support was relatively high (M + 1SD), the indirect effect was not significant (*β* = -0.064, BootSE = 0.031), and the bootstrap 95% CI of the indirect effect did not contain 0. At the same time, a comparison of the mediating effects under different levels of social support revealed that the mediating effects of nursing students’ career adaptability were significantly different (*β* = 0.139, BootSE = 0.041), and the bootstrap 95% CI of the indirect effect did not contain 0. Therefore, hypothesis 4 was supported.

**Table 4:** The indirect effect under different social support levels

Social support level	Effect size	BootSE	95%CI
Effect1(M + 1SD)	-0.064	0.031	-0.124~-0.001
Effect2(M-1SD)	-0.203	0.046	-0.294~-0.115
Difference(Effect1-Effect2)	0.139	0.041	0.056 ~ 0.220

## Discussion

Currently, while many studies have explored the antecedents influencing nursing students’ professional commitment [5, 7, 53], limited attention has been given to the impact of perceived work dirtiness on nursing students’ professional commitment and the underlying mechanisms of this relationship [31]. This study, which draws on career construction theory, successfully elucidates the inherent interaction mechanisms among nursing students’ perceived work dirtiness, career adaptability, professional commitment, and social support. The findings indicate that (a) nursing students’ perceived work dirtiness significantly negatively predicts their professional commitment; (b) perceived work dirtiness affects professional commitment through the partial mediating role of career adaptability; and (c) social support among nursing students moderates the relationship between perceived work dirtiness and career adaptability. Additionally, (d) the indirect impact of perceived work dirtiness on professional commitment through career adaptability is moderated by social support.

Previous research has revealed a significant negative impact of professional stigma on nursing and hotel management students’ professional commitment [27, 54]. The current study extends previous findings, demonstrating that perceived work dirtiness anticipates a negative impact on the professional commitment of nursing students. Notably, in comparison to professional stigma, perceived work dirtiness more intuitively expresses individuals’ feelings toward their profession [55]. Therefore, investigating the relationship between perceived work dirtiness and professional commitment is necessary for distinguishing between the concepts of perceived work dirtiness and professional stigma, as suggested by Kraus [56]. It also contributes to a more comprehensive understanding of the importance of individual subjective experiences in shaping professional commitment. Furthermore, this study posits that the negative impact of perceived work dirtiness on nursing students’ professional commitment is associated with two factors. On the one hand, individuals inherently have a predisposition to avoid dirt and impurities [57]. When nursing students perceive that their future career will entail physical dirtiness, motivation to engage in the nursing industry decreases, subsequently influencing individuals’ willingness to remain devoted to a profession [31]. On the other hand, societal culture plays a crucial role in shaping professional commitment. As pointed out by Pang, traditional Confucian teachings suggest that “virtuous children should not be soldiers, servants, or laborers.” [58]. In the context of Eastern culture, biases associated with the nursing profession not only directly impact nursing students but also indirectly affect their parents and relatives [59], conflicting with China’s culture of filial piety. This reluctance to engage in the nursing industry decreases professional commitment.

The findings of this study indicate that career adaptability mediates the relationship between perceived work dirtiness and professional commitment. While previous research has predominantly focused on the protective factors of career adaptability, emphasizing its positive influence on favorable career outcomes, it has overlooked the investigation of risk factors. Only a handful of studies, exemplified by Maggiori’s research, have highlighted that negative career prospects and work-related stress can disrupt the career construction process [39]. Grounded in career construction theory, our study explicitly posits that perceived work dirtiness leads to a reduction in career adaptability among nursing students, subsequently diminishing their professional commitment. This not only provides further validation for Maggiori’s findings but also addresses a critical gap in the current research regarding the negative aspects of the career construction process. However, in this study, career adaptability serves only as a partial mediator. This could be attributed to the relatively stable nature of career adaptability as a psychological concept [35], while the impact of perceived work dirtiness on career adaptability requires an extended period for development. Consequently, researchers are encouraged to identify additional potential mediating variables to achieve a comprehensive understanding of the enduring effects of perceived work dirtiness on professional commitment.

In this study, social support is considered a moderating factor in the relationship between perceived work dirtiness and career adaptability. Among nursing students with lower levels of social support, a stronger correlation was observed between perceived work dirtiness and career adaptability. Conversely, in groups with higher social support, the correlation between perceived work dirtiness and career adaptability weakens. This is likely because nursing students with high social support demonstrate an ability to recover from adversity rapidly [60, 61], aiding them in overcoming discomfort arising from perceiving their professional as dirty work. Consequently, this ability contributes to a reduction in the negative impact of perceived work dirtiness on career adaptability [62]. These findings align with the career adaptation model. Importantly, this study revealed that social support moderates the mediating role of career adaptability. In nursing student groups with low social support, the indirect effect of perceived work dirtiness on professional commitment is significant. As the social support of nursing students increased, the negative indirect effect of perceived work dirtiness on professional commitment gradually weakened. This suggests that high social support can mitigate the negative impact of perceived work dirtiness on nursing students’ career adaptability, thereby preventing a decline in professional commitment. This finding is consistent with the conclusion of Li, who indicated that social support plays an important role in the development of nursing students’ career abilities [16]. Hence, we emphasize the need for nursing educators to recognize the crucial role of social support in enhancing individual career adaptability and career well-being, enabling the development of more effective educational strategies and support measures.

### Limitations and implications

Methodologically, first, the cross-sectional design imposes constraints on establishing causal relationships. Future research should consider employing longitudinal methods or experimental designs for a more in-depth understanding of the relationship between perceived work dirtiness and professional commitment. Second, the study’s reliance on self-report measures introduces a potential bias due to social desirability. To gain a more comprehensive understanding of the impact of perceived work dirtiness on nursing students’ professional commitment, external assessments or qualitative interviews could be integrated into future investigations. Finally, given the potential variations in regional healthcare environments, the sample is limited to three universities in different cities in China, possibly limiting its external validity.

From a theoretical perspective, first, this study addresses the impact of perceived work dirtiness on professional commitment among nursing students, implying that our findings can only offer insights into nursing education. However, we did not directly measure key indicators of future nursing workforce sustainability, such as career choices. Therefore, exploring the relationship between perceived work dirtiness and career choices in future research would be a meaningful research topic. Additionally, once nursing students transition into professional nurses, how will their perceived work dirtiness impact them? Addressing this question requires future researchers to conduct further investigations, focusing on nurses as research subjects, to advance our understanding of occupation studies. Second, the results suggest that career adaptability plays only a partial mediating role, indicating the potential existence of other yet undiscovered mediating effects. Therefore, future research could delve into the intrinsic connections between perceived work dirtiness and professional commitment from alternative theoretical perspectives. Finally, this study predominantly examines societal-level variables—the moderating role of social support—without considering the potential moderating effects of variables at other levels in the relationship between perceived work dirtiness and professional commitment. Future research may explore individual- and school-level variables (such as attribution styles, personality traits, and institutional career support) to investigate other boundary conditions influencing the impact of perceived work dirtiness on professional commitment.

Despite these limitations, this study has significant theoretical and practical implications. Theoretically, the application of career construction theory provides a novel lens for comprehending the impact of perceived work dirtiness on professional commitment. The identification of career adaptability and social support as protective elements for professional commitment enriches our understanding of the relationship between perceived work dirtiness and professional commitment. From a practical perspective, the current research underscores that perceived work dirtiness might erode professional commitment through career adaptability and that social support plays a moderating role in this dynamic process. Thus, healthcare institutions should prioritize standardizing nursing work processes and leveraging advanced medical technologies to minimize direct exposure to dirty work environments. Simultaneously, initiatives should be taken to safeguard nurses’ rights in nurse‒patient relationships. Nursing educators are encouraged to integrate career education into students’ academic routines, promote proactive exploration of career interests and incorporate career adaptability training into professional courses. Additionally, gradually intensifying task complexity during nursing internships and providing targeted career-related information can boost nursing students’ career confidence and readiness. Establishing online platforms for student communication and instituting mentoring systems can act as conduits for social support, facilitating both career exploration and enhancing professional commitment among nursing students.

## Conclusion

This study revealed that perceived work dirtiness exerts an indirect impact on professional commitment through the mediating role of career adaptability. Additionally, social support was identified as a moderator in the relationship between nursing students’ perceived work dirtiness and career adaptability. Moreover, the protective effect of social support on nursing students’ career adaptability suggests a potential avenue for mitigating the adverse influence of perceived work dirtiness on professional commitment. Considering these findings, it is recommended that nurse educators implement targeted interventions. This may include the development of career adaptability courses for nursing students and the provision of platforms for peer support aimed at facilitating a smooth transition into the nursing profession.

## Data Availability

The datasets analyzed during the current study are available from the corresponding author upon reasonable request.
